# The Treatment of Malignant Tumors by Electrolysis

**Published:** 1869-09

**Authors:** W. Neftel

**Affiliations:** New York


### The Treatment of Malignant Tumors by Electrolysis.

BY W. NEFTEL, M. D., NEW YORK.

Although I am preparing for publication my researches on the continuous galvanic current, where remarkable illustrations of the electrolytic treatment will be given in extenso, I consider it my duty to give at once a preliminary account of the following case, which may contribute to save the lives of many sufferers deemed incurable.

Hon. Th. T. D——, a highly accomplished gentleman, 58 years old, consulted last year several celebrated surgeons in London and Paris, (amongst others, Nelaton,) with regard to a tumor in the left mammary region. They all advised him not to undergo any surgical operation, as they considered the tumor a malignant one, the removal of which would only hasten the fatal termination of the undoubtedly constitutional disease. The patient, nevertheless, insisted upon the extirpation of the tumor, and our great surgeon, Dr. Marion Sims, quite successfully performed the operation in Paris. Soon after the cicatrization of the wound, however, the axillary glands of the same (left) side began to enlarge, and in January last presented a tumor of the size of an egg, consisting of a conglomeration of enlarged and indurated glands. Dr. Sims again extirpated this second tumor, the microscopical appearance of which was that of a real cancer (carcinoma of the axillary glands.) The specimen was presented to the New York Pathological Society and examined by distinguished histologists.* The wound this time healed very slowly, as it was accompanied by dangerous complications, an extensive erysipelas, high fever (107.8° Fahr.), rigors and delirium. Scarcely had the wound healed, when a new scirrhous tumor began to grow in the right mammary region, and very soon attained the size of an orange, or more. It now became evident that another surgical operation would be useless, for it could only call forth, as before, an immediate relapse, and perhaps in a more dangerous locality. As nothing remained to save the patient, who was perfectly aware of his condition, and whose constitution was broken down, I proposed the electrolytic treatment, expecting, as the best result, merely the local destruction and absorption of the tumor; for, in the present state of our knowledge, I could not have entertained any hope of producing by electrolysis the least favorable change in the constitutional disease.

On the 27th of April, and the 4th and 7th of May, in the presence of Drs. Metcalf, Nott, and B. Howard, I performed the electrolysis by means of the large apparatus of Kruger and Hirschman, with elements of Siemens, subdividing, at the second and third operation, the cathode into three and four branches, connected with the needles by serres-fines. The latest improvements of the apparatus afforded the possibility of gradually increasing the quantity of the current, without interrupting the circuit, and of diminishing it in the same way, so that the circuit was broken only by the extraction of the last needle. Not a drop of blood escaped. The first operation lasted two minutes, using ten elements; the second five minutes, with twenty elements; and the third ten minutes, with thirty elements. After the operation the tumor increased considerably in size, but became softer and more elastic. No febrile or other local or constitutional symptoms followed. On the contrary, the patient who before was weak, anæmic and cachetic, began to gain strength and flesh; the tumor at the same time diminishing slowly but constantly. A month after the first sitti g the tumor was found a great deal softer and smaller; at the end of the second month it had almost disappeared, and a fortnight later no trace of it remained. The general condition of the patient is now in all respects excellent, and new deposits can nowhere be detected. In his last letter he writes to me as follows: "I am not able to discover any new deposits anywhere, nor would the tumor in the right breast be detected by any ordinary observer. I hope the old devil who took lodgings there and was ejected, took all his baggage with him."†

The above related case presents the following points of interest:

1. The patient has been examined by a number of celebrated physicians in Europe and America, who have all considered him affect-

---

*Medical Record, March 1, 1869, No. 73, p. 17

†The patent has since returned to the city, and been seen by some of the physicians above named.

ed by a constitutional cancerous disease; and the extirpated tumors, being real cancers, have proved the correctness of the diagnosis.

2. The described case brings me to the conclusion that the electrolysis must be considered not only as a local agent, as thinks Althaus,* but as one capable of modifying, and even curing, the constitutional diathesis. I explain it in the following way: It has already been established, by experimental researches, that the electric current affects powerfully all protoplasmatic structures.† Hence it is possible and probable that the cells (which have to be considered as bearers of the contagion and the cause of the generalization of the disease) get their protoplasma altered in such a way by electrolysis, as to lose its specific infectious properties, and make it incompatible with the existence and propogation of the cancerous new formation.

3. Finally, this is the first authentic case of cure of a real cancer in a subject affected with constitutional diathesis. I think that if Althaus, to whom we are indebted for the improved electrolytic method, did not succeed in curing a single case of malignant tumor,‡ it is owing only to the imperfection of the apparatus with which he works. I have had one like it imported from London, and have ascertained, by the feeble deflexion of the needle of my galvanometer, and by the weak muscular reaction it produces, that Althaus' apparatus generates a very small current-quantity. This explains also why he is obliged to have recourse to so numerous and prolonged sittings (half an hour,) whilst, with the excellent apparatus I am in the habit of using, incomparably better results can be obtained in a much shorter period.

With regard to non-malignant tumors, I will give in my next paper an account of what I have attained by electrolytic treatment. Especially the soft tumors, nævi, etc., yield very rapidly to it. A large goitre of eighteen years' standing has completely disappeared in the course of two months. So far as I can judge from my experiments on animals (rabbits,) the electrolytic treatment of varicose veins and aneurisms promises to be highly successful. Examining microscopically the thrombi, I could repeatedly convince myself, in opposition to the assertions of Tschaussoff,§ that the organization of a thrombus really does take place, a fact which had been already experimentally demonstrated by the classical researches of Virchow,* as far back as 1846. Again, it is not difficult to follow up the gradual transformation of the colorless blood-corpuscles into connective tissue-corpuscles, which was likewise accepted by Virchow.

But the most surprising effect can be produced, by the electro-

---

* On the Electrolytic Treatment of Tumors. London. 1867, p. 10.

† Vide Kuhne: Lehrbuch der physiologischen Chemie, p. 333.—Golubew: Wirkung electrischer Schlage auf die farblosen Formbestandtheile des Blutes. Cent. f. med. Wiss. 1869- No. 5.

‡ Medical Times and Gazette. 1868, p. 469.

§ Archive for klin. Chirurgie- xi. 184.

* Gesammelete Abhandl., p- 323.

lytic treatment, on organic strictures of the urethra. The only case I have had is a gentleman who is yet under my observation. He has been suffering for about ten years from organic strictures impermeable even for the thinnest bougies. He told me that, though he had been under the care of many distinguished surgeons, no one could ever succeed in introducing a catheter into his bladder. On the 20th July I introduced a French catheter, No. 3, up to the principal stricture, situated in the prostatic part of the urethra, the prostate itself being enormously enlarged, and by a very simple contrivance, directed the electrolytic action of the negative pole upon the stricture during two minutes. Immediately, to my great astonishment, the catheter passed within the bladder, and an immense quantity of turbid and decomposed urine was discharged. Since this the patient has been able to pass urine easier than he has ever done before. On the 24th July I repeated the operation with the same result, but using catheter No. 6 of the French scale; and I can now introduce Nos. 8 and 10 without resorting to electrolysis. So far as I can ascertain, the prostate itself does not seem to be enlarged any longer.

In spermatorrhœa this mode of treatment cannot be surpassed. I have had several cases of inveterate spermatorrhœa, which all yielded to a single or to repeated electrolytic treatment of the prostatic part of the urethra. I am sure that those who have once tried this method, will find it far superior to all the others, which are comparatively tedious and uncertain.

The first discoverer of the electrolytic treatment was Crussel,‡ of St. Petersburg, Russia. Already in 1839 he demonstrated experimentally the different effects produced by the different poles, and used electrolysis in the treatment of strictures, exudations, tumors, and ulcers. A number of others followed him, amongst whom one of the most successful is undoubtedly my friend Dr. Moritz Meyer,§ of Berlin. Dr. Althaus has quite recently improved the method and shown the great importance of the negative pole in the treatment of tumors. Certainly every observer will agree with him that electrolysis, besides annihilating the pain, acts in a threefold manner,‖ viz.: 1. Through mechanical disintegration of the tissues by the nascent hydrogen; 2. Through the dissolving action of the accumulated free alkali (potash, soda and lime); 3. Through the local modification of nutrition (by means of the vasomotor nerves) of the parts brought under the immediate influence of the current. To these local effects I can now add, from my own experiments and observations, the constitutional effect of electrolysis, which latter especially makes this method invaluable in many hitherto incurable diseases. One of its great advantages is that it is never followed by

---

‡ Crussel: Die electrolytische Heilmethode.—Medic. Zeitung Russlands. 1847–48.

§ Moritz Meyer: Die Electricitat in ihrer Anwendung auf practische Medicin. Aufl. 1868, p. 405, 407.

‖ Op. cit., p. 441.

inflammation, suppuration, sloughing, or other disturbances, and that the patient can continue his usual occupation and mode of life.

The electrolytic treatment is called to open a large field of surgery, and will be applied very soon to a variety of surgical diseases, to the advantages of the profession and the benefit of suffering humanity. The surgeon now, besides his biological knowledge and the use of his mechanical appliances, will acquire and appropriate to himself the knowledge of physics, electro-physiology, and the management of the complicated galvanic apparatus.—*Med. Record.*

